# Integrated Molecular and Histological Insights for Targeted Therapies in Mesenchymal Sinonasal Tract Tumors

**DOI:** 10.1007/s11912-024-01506-9

**Published:** 2024-02-20

**Authors:** Cosima C. Hoch, Leonard Knoedler, Samuel Knoedler, Ali Bashiri Dezfouli, Benedikt Schmidl, Anskar Trill, Jennifer E. Douglas, Nithin D. Adappa, Fabian Stögbauer, Barbara Wollenberg

**Affiliations:** 1 Department of Otolaryngology, Head and Neck Surgery, School of Medicine and Health, Technical University of Munich (TUM), Ismaningerstrasse 22, 81675 Munich, Germany; 2 Department of Surgery, Division of Plastic Surgery, Yale School of Medicine, New Haven, CT USA; 3 Division of Plastic and Reconstructive Surgery, Massachusetts General Hospital, Harvard Medical School, Boston, MA USA; 4 Institute of Regenerative Biology and Medicine, Helmholtz Zentrum Munich, Munich, Germany; 5 Central Institute for Translational Cancer Research, Technical University of Munich (TranslaTUM), Department of Radiation Oncology, Klinikum rechts der Isar, Munich, Germany; 6 Department of Otorhinolaryngology, Head and Neck Surgery, University of Pennsylvania, Perelman School of Medicine, Philadelphia, PA USA; 7 Institute of Pathology, School of Medicine and Health, Technical University of Munich (TUM), Munich, Germany

**Keywords:** Mesenchymal sinonasal tract tumors, Sinonasal tract angiofibroma, Sinonasal glomangiopericytoma, Biphenotypic sinonasal sarcoma, Skull base chordoma, Targeted therapy

## Abstract

**Purpose of Review:**

This review aims to provide a comprehensive overview of mesenchymal sinonasal tract tumors (STTs), a distinct subset of STTs. Despite their rarity, mesenchymal STTs represent a unique clinical challenge, characterized by their rarity, often slow progression, and frequently subtle or overlooked symptoms. The complex anatomy of the sinonasal area, which includes critical structures such as the orbit, brain, and cranial nerves, further complicates surgical treatment options. This underscores an urgent need for more advanced and specialized therapeutic approaches.

**Recent Findings:**

Advancements in molecular diagnostics, particularly in next-generation sequencing, have significantly enhanced our understanding of STTs. Consequently, the *World Health Organization* has updated its tumor classification to better reflect the distinct histological and molecular profiles of these tumors, as well as to categorize mesenchymal STTs with greater accuracy. The growing understanding of the molecular characteristics of mesenchymal STTs opens new possibilities for targeted therapeutic interventions, marking a significant shift in treatment paradigms.

**Summary:**

This review article concentrates on mesenchymal STTs, specifically addressing sinonasal tract angiofibroma, sinonasal glomangiopericytoma, biphenotypic sinonasal sarcoma, and skull base chordoma. These entities are marked by unique histopathological and molecular features, which challenge conventional treatment approaches and simultaneously open avenues for novel targeted therapies. Our discussion is geared towards delineating the molecular underpinnings of mesenchymal STTs, with the objective of enhancing therapeutic strategies and addressing the existing shortcomings in the management of these intricate tumors.

## Introduction

Sinonasal tract tumors (STTs), although relatively uncommon, represent major clinical challenges in the field of head and neck medicine, comprising both benign and malignant entities. Accounting for approximately 5% of head and neck tumors, these neoplasms have an annual incidence rate of 0.5 to 1.0 per 100,000 individuals [1]. Their development is associated with environmental risk factors, including exposure to industrial by-products, such as metals, textiles, leather, and wood dust [2].

The presentation of STTs, which can include symptoms, such as purulent nasal discharge, epistaxis, and nasal obstruction, is often non-specific and insidious, leading to delayed diagnosis and varying prognoses. While benign tumors may present less aggressive behavior, malignant STTs have a more dire prognosis, with 5-year survival rates dropping to 20% in advanced stages [3••]. The complex anatomy of the sinonasal region, which includes vital structures, including the orbit, brain, and cranial nerves, adds to the challenges in treating these tumors [4]. Surgical resection remains the primary treatment objective, but complete tumor removal is often challenging due to these anatomical constraints [5]. Consequently, adjuvant therapies (i.e., radiotherapy and chemotherapy) play a crucial role in managing residual disease and improving local control.

Advances in molecular diagnostics, particularly next-generation sequencing (NGS), have revolutionized our understanding of STTs [6]. These technologies have enabled more precise tumor subtyping, as exemplified by the *World Health Organization's classification system*, which categorizes STTs based on their histological and molecular markers [7••]. This system distinguishes different tumor types, including hamartomas, respiratory epithelial lesions, and mesenchymal tumors.

Our review specifically focuses on mesenchymal STTs, a subset of STTs that includes entities, such as sinonasal tract angiofibroma, sinonasal glomangiopericytoma, biphenotypic sinonasal sarcoma, and skull base chordoma. These tumors are particularly noteworthy due to their unique histological and molecular profiles, which not only challenge conventional diagnostic and treatment strategies but also present opportunities for the development of targeted therapies. By exploring the molecular underpinnings of mesenchymal STTs, we aim to highlight the potential for more precise and effective treatment modalities, addressing a critical gap in the current management of these complex tumors.

## Sinonsal Tract Angiofibroma

Sinonasal tract angiofibroma (STA), also known as juvenile nasopharyngeal angiofibroma, is a tumor that, while histologically benign, exhibits a high degree of vascularity [8]. As the most common non-cancerous tumor in the sinonasal region, STA accounts for up to 0.5% of all head and neck tumors [9]. STA predominantly affects male adolescents, typically in their second decade of life [10]. The tumor carries a significant risk of local recurrence, estimated at 40%, especially if it is not completely excised [11]. The exact etiology of STA remains a subject of debate, with theories ranging from vascular malformations to branchial remnants [12]. Notably, familial predisposition is well documented, with individuals carrying the familial adenomatous polyposis (FAP) gene being 25 times more likely to develop STA [13]. Treatment primarily involves preoperative embolization followed by surgical resection based on thorough clinical and radiological assessments, typically avoiding preoperative biopsies [14, 15]. Although malignant transformation in STA post-radiotherapy has been reported, it is still recommended as adjunctive therapy in cases of incomplete tumor removal, unresectable tumors, or extensive intracranial extension [11, 16]. For recurrent cases, chemotherapy options, such as doxorubicin and dacarbacine, ought to be considered despite limited long-term experience regarding its therapy success [17].

### Histopathological Characteristics of STA

In STA, characteristic histological features include the development of unevenly distributed vascular channels in a fibrous matrix. This matrix is composed of varying amounts of collagen fibers and cells that are typically plump and either spindle or stellate in shape [18]. A consistent finding across studies is the presence of CD34 in all vascular cells, as revealed by immunohistochemical analysis [19–21]. Starlinger et al. further underscored the diverse vascular structures present in STA [22]. The high expression levels of laminin alpha2 found in the vasculature of STA suggest that these vessels may be at an early developmental stage of the tumor [22]. This finding substantiates the hypothesis that remnants of the first branchial arch artery's plexus play a significant role in the formation of the vascular component of this tumor [12].

### The Molecular Landscape of STA

**Fig. 1:**
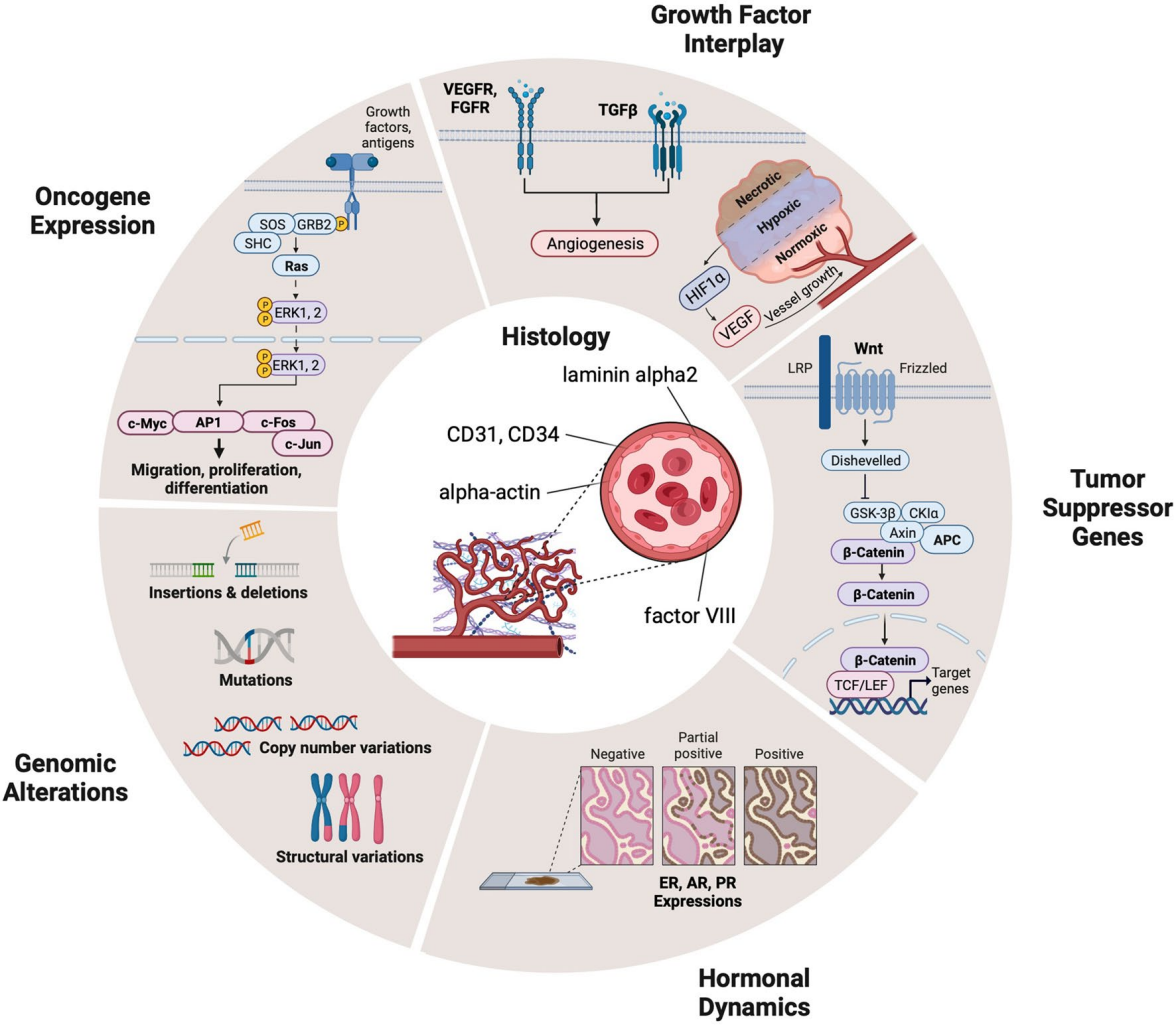
Intricate molecular and histological network of STA. VEGFR, vascular endothelial growth factor receptor; FGFR, fibroblast growth factor receptor; TGFβ, transforming growth factor-β; HIF1α, hypoxia-inducible factor 1α; GSK-3β, glycogen synthase kinase-3β; LRP, lipoprotein receptor-related proteins; CK1α, casein kinase 1α; APC, adenomatous polyposis coli; TCF/LEF, T-cell factor/lymphoid enhancer factor; ER, estrogen receptor; AR, androgen receptor; PR, progesterone receptor; ERK, extracellular signal-regulated kinase; CD31, platelet endothelial cell adhesion molecule (Figure created using BioRender, Toronto, ON, Canada)

Over the past three decades, substantial progress has been achieved in understanding the molecular characterization of STA. A comprehensive review of the literature suggests a structured categorization of these findings for better clarity and analysis. This categorization includes genomic alterations, the role of tumor suppressor genes, the expression of oncogenes, the dynamics of growth factor interactions, and hormonal influences. Figure 1 illustrates this intricate molecular and histological network, delineating the interplay between these various factors.

#### Genomic Alterations

Molecular genetic techniques, such as loss of heterozygosity (LOH) analysis, Fluorescence In Situ Hybridization (FISH), Comparative Genomic Hybridization (CGH), and Real-Time Quantitative Polymerase Chain Reaction (RT-qPCR) have revolutionized the detection of chromosomal alterations in STA. The methods have been instrumental in identifying regions harboring potential oncogenes or tumor suppressor genes. A landmark study by Schick et al. utilized CGH and FISH to identify genetic imbalances in STA, finding an extra chromosome X and loss of chromosome Y, along with further chromosomal gains (8q12-q22) and losses (17, 19p, and 22q) [23, 24]. In addition, they noted chromosomal aberrations on several chromosomes, including frequent gains and losses, and amplification of the AUKRA (STK15) and MDM2 genes, which may contribute to chromosomal instability [25]. Brunner et al. documented diverse chromosomal abnormalities in STA, including frequent gains and a complete loss of the Y chromosome [26]. Employing CGH, Heinrich et al. also reported frequent DNA gains in STA [27]. Gene expression analyses have revealed a positive correlation between endothelial and stromal components for genes such as ASPM, CDH1, CTNNB1, FGF18, and SUPT16H [28]. Calanca et al. identified significant alterations in gene expression, with increased expression of COL4A2 and LAMB1 and decreased expression of BCL2 and RAC2, as assessed by RT-qPCR [29•].

#### Tumor Suppressor Genes

The observed higher incidence of STA in individuals with FAP suggests a potential genetic link between these conditions. The adenomatous polyposis coli (APC) gene, located on chromosome 5q21, is well known to cause FAP [30••]. The APC gene product is a crucial regulator of beta-catenin, a key element in cell adhesion and the Wnt signaling pathway [31].

However, research investigating APC mutations in STA, yielded varying results. Ferouz et al. and Guertl et al. found no APC mutations in their analyses of five and eleven STA cases, respectively [13, 32]. Conversely, significant beta-catenin gene transcript expression was observed in 75% of STA, with notable nuclear accumulation in the stromal cells of these tumors [33]. This was further supported by findings of nuclear staining of beta-catenin in both sporadic and familial STA, with altered APC expression particularly noted in FAP-associated STA [34]. Additionally, variations in Wnt pathway gene expressions, such as reduced WNT5A and increased WNT5B, were identified, suggesting alternative mechanisms of Wnt pathway involvement in STA pathogenesis [29•]. Zhang et al. reported strong beta-catenin expression in STA compared to nasal polyps [35], and similar findings were confirmed by Rippel et al. and Pandey et al. in their studies on stromal and endothelial cells of STA [36, 37]. However, the expression of beta-catenin in STA has been found to be inconsistent, challenging the assumption of its universal amplification in STA [38].

Separately, the tumor suppressor gene TP53, known for its role in cell growth regulation, has also been a subject of interest in STA research. Studies have reported different TP53 mRNA expression levels in STA, with some cases showing increased expression, while others found gene losses without mutations [39, 40]. Intriguingly, lower TP53 mRNA expression was associated with skull-base involvement, whereas a higher expression correlated with lateral extension [41].

#### Oncogene Expression

The c-myc gene, encoding a phosphoprotein essential for cellular growth, proliferation, and apoptosis, exhibits potent angiogenic effects and is commonly deregulated in malignancies [42•]. In STA, c-myc expression has shown mixed results, with some studies finding no significant differences from normal tissues, while others reported overexpression or loss in certain cases [40, 43, 44]. Notably, higher c-myc mRNA expression was associated with skull base involvement, whereas lower expressions correlated with lateral extension [41].

The c-kit protooncoprotein, a tyrosine kinase receptor, plays a central role in mesenchymal tumors and is a treatment target in specific cases. Its strong expression in STA stromal and endothelial cells, along with high mRNA expression, indicate its clinical relevance [35, 37, 44]. However, conflicting findings have also been reported, with one study detecting no c-kit expression in STA [45].

Regarding the ras gene, known for its involvement in cellular signal transduction, no mutations in key codons were found in STA [46]. Increased mRNA expression of ras, correlating with clinical characteristics, such as intraoperative hemorrhage, tumor volume, skull base extension, and recurrence potential, has been described by Pandey et al. [37, 41].

The fos family proteins (c-fos, FosB, Fra-1, and Fra-2), which, together with the Jun protein, form the AP-1 transcription factor complex, influence cell proliferation, death, differentiation, and inflammation [47]. The c-fos gene, known for its oncogenic potential, is frequently overexpressed in tumors and showed increased expression in 14% of STA cases [40].

Finally, the Her-2/neu gene, which has been linked to various tumors, showed no amplification in a small-scale STA study [39].

#### Angiogenesis and Growth Factor Interplay

The molecular characteristics of STA are strongly influenced by a network of growth factors that are essential for tumor development and angiogenic responses. In this regard, vascular endothelial growth factor (VEGF), a prominent proangiogenic factor in tumor biology, plays a key role [48]. The role of VEGF in STA has been extensively studied, with findings indicating that its significant expression is associated with enhanced cell proliferation and increased blood vessel density [49–51]. This is in line with Mishra et al.’s findings of increased mRNA expression of VEGF in STA, correlating with clinical variables, such as intraoperative hemorrhage, tumor volume, skull base extension, and recurrence [41]. Transcription of VEGF genes is activated under hypoxic conditions by an inhydroxylated hypoxia-inducible factor 1α (HIF-1α) [52]. Song et al. found higher HIF-1α expression in recurrent STA cases, suggesting its potential as a prognostic marker for recurrence [53].

Equally important are the fibroblast growth factors (FGF and bFGF), which are involved in angiogenesis and tissue development [54]. Increased expression of these factors has been reported in STA, implying their role in the disease pathogenesis [41, 50, 55]. This is corroborated by the findings of Safhi et al., who noted significant upregulation of fibroblast growth factor receptor (FGFR)3/4 genes in STA patients, with a more pronounced association among smokers [56•]. Jones et al. also documented an upregulation of FGFR2 in patient STA sections [51]. The importance of FGFR signaling is further underlined by studies showing that STA fibroblast proliferation, migration, and invasion can be inhibited by blocking FGFR signaling pathways, as exemplified by the efficacy of AZD4547 treatment in vitro [57].

Transforming growth factor (TGF)-beta 1, secreted by fibroblasts, macrophages, and endothelial cells, is another relevant factor in STA. It plays a crucial role in the regulation of the cell cycle, extracellular matrix production, and angiogenesis [58]. Its significant presence in STA has been documented in multiple studies, pointing to its importance in stromal and vessel growth [40, 50, 59, 60].

While other factors like bone morphogenic proteins from the TGFb superfamily, platelet-derived growth factor (PDGF), insulin-like growth factors (IGFs), and nerve growth factor (NGF) have been studied in STA, their specific roles are not as clearly defined [35]. The variable expression levels of IGFs in STA hint at their possible involvement in tumor growth [61], and the role of NGF in vascular growth in STA represents an intriguing area for further research [35].

#### Hormonal Dynamics

The development of STA is believed to be hormonally driven. This hypothesis is backed up by the relatively high prevalence of STA in young men and its onset typically during the years of sexual maturation [62]. Initial research in this area suggested that imbalances in sex hormones might underlie the occurrence of STA [63–65]. Martin et al. reported that patients with STA often experienced delayed sexual maturation, and it was noted that the size of the tumor tended to decrease following the onset of secondary sexual characteristics [64].

Further studies on the hormonal aspects of STA confirmed the impact of sex hormones on its progression [66–69]. For instance, estrogen therapy, which was widely used in the 1960s and 1970s to reduce tumor size and surgical bleeding, is no longer a standard practice. This change is attributed to the inconsistent results and potential side effects of the therapy [70, 71]. Additionally, the hypothesis that testosterone could exacerbate tumor growth led to the exploration of treatments with anti-androgenic agents, such as cyproterone acetate and flutamide. However, these treatments showed inconsistent efficacy in reducing the growth of STA cells and the overall tumor size [71–73, 74••].

Analyses of hormone receptors in STA tissues yielded divergent results. While some studies found no presence of estrogen receptors (ERs) in STA tissues [65, 66], others detected androgen receptors (ARs) but no ERs or progesterone receptors (PRs) [75, 76]. Hwang et al. observed ARs presence in 18 out of 24 STA cases, with immunostaining evident in stromal and endothelial cells [77]. In contrast, Gatalica found no ERs or progesterone receptors in 8 STA cases and 8 nasal turbinate control samples, with only minor nuclear AR immunoreactivity in some endothelial and stromal cells of both tumor and normal tissues [78]. This aligns with findings of Pandey et al., who also failed to detect AR expression in STA samples [37]. Saylam et al. conducted an immunohistochemical analysis of 27 STA samples and discovered ER presence in 7.4% of cases and PRs in 33.3% [60]. In this context, it is also worth mentioning that Brentani et al. reported a correlation between the presence of ARs and PRs, and a higher density of endothelial and fibroblast cells in STA [67]. Moreover, recent studies have suggested that ER-α, alone or in combination with heat shock protein (Hsp)90, might serve as an indicator for predicting tumor recurrence [79, 80]. These findings indicate that hormone receptor stability, potentially influenced by Hsp90, plays a significant role in STA development.

Despite the evidence pointing to hormonal imbalances in STA patients and the detection of ARs and/or ERs in STA tissues, no consistent changes in serum hormone levels have been found. This discrepancy leaves the hormonal impact on STA a subject of ongoing debate in the scientific community.

### New Horizons in STA Treatment

Research regarding new therapeutic strategies for STA is still in its infancy, with only a single clinical trial currently registered on ClinicalTrials.gov (NCT05549167). One key aspect currently under investigation is the role of mammalian target of rapamycin (mTOR) signaling in the growth and vascularization of STA. Sirolimus, an mTOR inhibitor, has emerged as a potential therapeutic agent [81••]. The trial is intended to fill a knowledge gap, as available data on the efficacy and safety of Sirolimus in STA are scarce and primarily based on few clinical cases. The primary aim of the study is, therefore, to evaluate the effectiveness and safety of Sirolimus, especially in young patients with primary or recurrent STA.

In parallel to pharmacological interventions, a novel diagnostic and potentially therapeutic approach targeting diverse somatostatin receptor subtypes (SSTRs) in STA stromal cells is being explored. This has led to the utilization of advanced imaging methods, such as ^68^Ga-DOTANOC PET/CT, which binds to multiple SSTRs, improving the accuracy of STA imaging [82]. Recent evidence has demonstrated uniform DOTANOC uptake in all analyzed cases of STA, underscoring its potential in preoperative diagnostics and postoperative assessments [83••]. This finding also highlights the potential use of radionuclide-based therapies targeting SSTRs for more specific treatment. However, current data indicates that the maximum standardized uptake values in STA are lower than in the pituitary gland [82]. Accordingly, the use of more potent radioactive methods for therapy is deemed hazardous at this stage.

## Sinonsal Glomangiopericytoma

Sinonasal glomangiopericytoma (SGP), also known as sinonasal-type hemangiopericytoma, is a rare vascular soft tissue neoplasm. Originating in the nasal cavity and paranasal sinuses, it represents less than 0.5% of all primary sinonasal tumors [84, 85]. SGP predominantly affects individuals in their 60s and 70s, showing a slight inclination towards the female population. The risk of recurrence post-treatment is relatively low, approximately 20%, typically manifesting within the first five years post-therapy [86]. The exact cause of SGP remains elusive, but different hypotheses suggest a link to increased blood vessel growth, potentially triggered by factors like trauma, pregnancy, hypertension, or corticosteroid usage [85]. The preferred treatment for SGP is endoscopic surgical excision [87, 88]. Recurrences are generally attributed to incomplete removal during initial surgery [84]. To reduce the likelihood of recurrence, adjuvant radiotherapy can be employed following surgery [89]. To date, no specific clinical trials have been conducted for SGP. While there are established clinical trials for hemangiopericytoma, it is important to note that SGP, with its unique pathological features, is different from solitary fibrous tumors, formerly known as hemangiopericytoma [90]. Therefore, in this review, we will not discuss clinical trials pertaining to hemangiopericytoma, but rather focus on the particularities of SGP.

### Histopathological Characteristics of SGP

SGP originates from pericyte cells surrounding capillaries [84]. SGP is characterized by a richly vascularized stroma, featuring vascular channels lined by a single layer of endothelial cells ranging from flat to cuboidal in shape [91]. The tumor’s cellular composition is predominantly oval or spindle-shaped cells with a “patternless” architecture with hyperchromatic nuclei, characterized by inconspicuous nucleoli and minimal eosinophilic cytoplasm [92]. Some areas of the tumor exhibit myxoid changes, presenting a gelatinous texture, and significant hyalinization around the blood vessels [92].

Immunohistochemically, SGP cells express nuclear beta-catenin, vimentin, smooth muscle actin (SMA), CD99, cyclin D1, and transducing-like enhancer of split 1 (TLE1) [90, 92–94]. Notably, cyclin D1 shows prominent nuclear expression in tumors that also express nuclear beta-catenin, suggesting a potential link between beta-catenin mutational activity and cyclin D1 overexpression in SGP pathogenesis [93]. The tumor does not typically stain for cytokeratin AE1/AE3, desmin, and nuclear signal transducer and activator of transcription (STAT)6 [90, 92, 94, 95]. Contrary to traditional viewpoints, the absence of CD34 reactivity, once thought to be a defining feature of SGP, has been challenged by Sangoi et al. due to high variability in CD34 expression and laboratory inconsistencies [86, 90, 93, 94, 96•, 97]. This may sensitize providers when using negative CD34 reactivity as a sole diagnostic criterion, especially in small tissue samples. Their findings advocate for a more inclusive diagnostic approach that employs a comprehensive panel of immunostains, rather than solely relying on CD34 [96•].

### CTNNB1 Mutations and Wnt Pathway Involvement in SGP

**Fig. 2:**
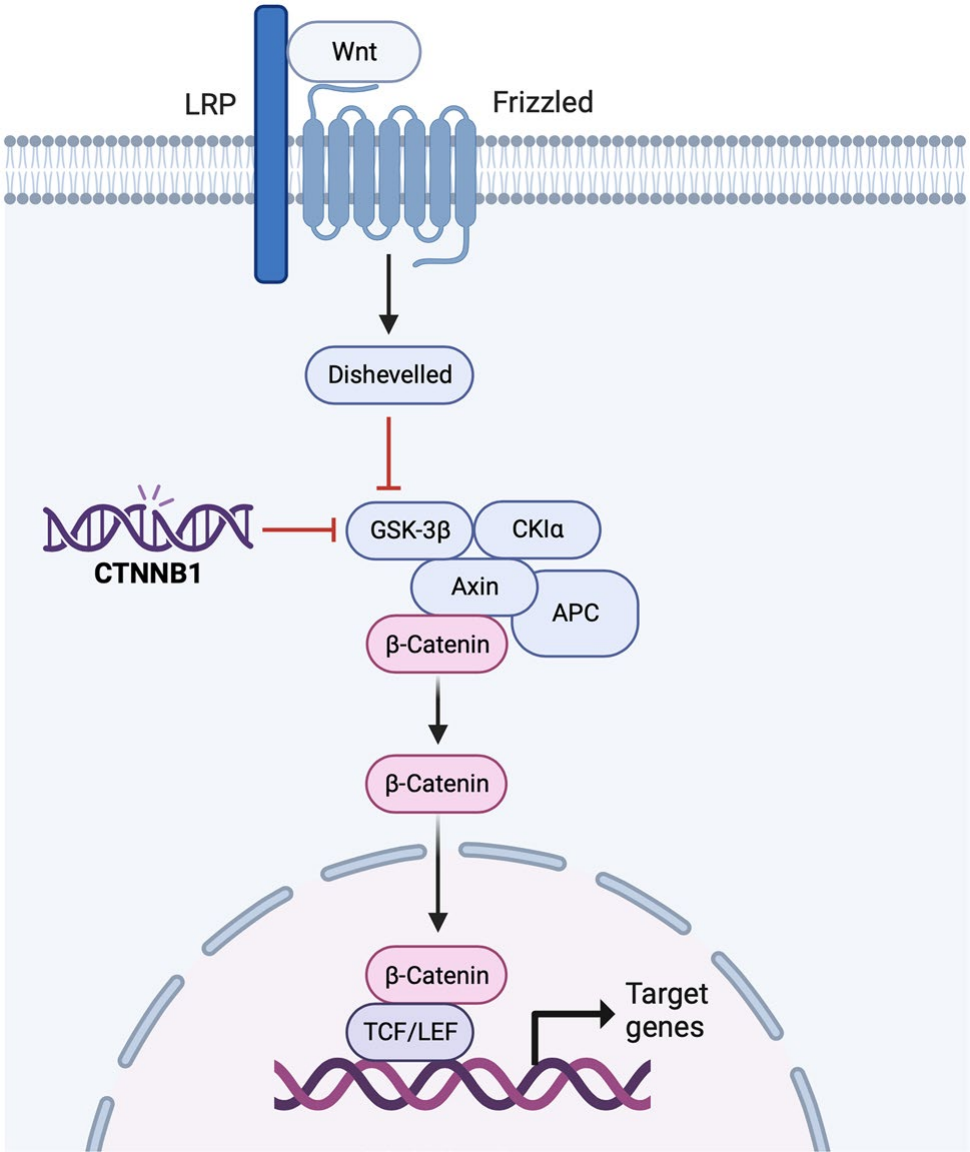
Glycogen synthase kinase-3β (GSK-3β) diminishes β-Catenin levels, a key player in the Wnt signaling pathway, by phosphorylating Serine/Threonine (Ser/Thr) sites at its N-terminal end. This leads to ubiquitination and subsequent breakdown by proteasomes. In sinonsal glomangiopericytoma (SGP), mutations in the CTNNB1 gene, which alter one of the vital Ser/Thr sites in the β-catenin’s GSK-3β region, lead to the stabilization of β-catenin. This, in turn, activates the β-catenin/T-cell factor/lymphoid enhancer factor (TCF/LEF) target genes. LRP, lipoprotein receptor-related proteins; CK1α, casein kinase 1α; APC, adenomatous polyposis coli (Figure created using BioRender, Toronto, ON, Canada)

The molecular pathogenesis of SGP remains partially understood, but recent advancements turned the focus on certain aspects. For instance, one significant discovery is the identification of CTNNB1 gene mutations and the consequent nuclear accumulation of beta-catenin in SGP, a feature distinct from the NAB2-STAT6 fusion typically associated with solitary fibrous tumors [93, 98, 99]. Research by Obeidin et al. further supports this understanding, revealing missense mutations in the CTNNB1 gene, specifically at the beta-catenin destruction complex recognition site, in four SGP cases [92]. These mutations predominantly involve a serine residue, impeding the phosphorylation and subsequent proteasomal degradation of beta-catenin. Consequently, there is an accumulation of beta-catenin in the nucleus, enhancing the transcription of Wnt pathway target genes, such as lymphoid enhancer binding factor 1, as highlighted in studies by Suzuki et al. (Fig. 2) [100]. Moreover, Lasota et al. have also identified mutations at these critical residues in SGP, reinforcing the role of beta-catenin pathway alterations in its pathogenesis [93]. While SGP shares histologic and immunohistochemical features with glomus tumors, including perivascular patterns and SMA expression, the MIR143-NOTCH gene fusion, commonly observed in glomus tumors, has not been detected in SGP [86, 101].

## Biphenotypic Sinonasal Sarcoma

Biphenotypic sinonasal sarcoma (BSNS) is an uncommon sinonasal malignancy, characterized by both neural and myogenic properties. Despite being a slow-growing and low-grade malignancy, BSNS exhibits a locally aggressive growth pattern [102]. The average onset age is between 50 and 53 years, with a female to male incidence ratio of approximately 2–3:1 [103, 104]. At diagnosis, around 20% of patients show bone invasion, particularly in the orbit (25%) and cribriform plate (10%) [102]. The long-term prognosis of BSNS remains unclear due to sparse research, but it is believed to have a higher five-year survival rate compared to other STTs. Literature suggests a recurrence rate of 32%-40%, occurring within 1 to 9 years post-treatment [105]. Given its slow-growing nature, surgical excision is the primary treatment for BSNS. If surgical margins are inconclusive or positive, adjunctive chemotherapy or radiation therapy may be considered [103, 105]. As of now, no clinical trials have been conducted for BSNS.

### Histopathological Characteristics of BSNS

Histologically, BSNS is characterized by uniformly elongated spindle-shaped cells, displaying minimal pleomorphism and low mitotic activity, alongside benign epithelial proliferation [106]. These features closely resemble those seen in cellular schwannomas or malignant peripheral nerve sheath tumors [107]. In some instances, BSNS may include larger cells with eccentric nuclei, prominent nucleoli, and brightly eosinophilic, fibrillary cytoplasm, occasionally showing cross-striations indicative of rhabdomyoblastic differentiation [108]. The histology of BSNS often displays a characteristic herringbone pattern [7••]. Additionally, a common observation includes the presence of a hemangiopericytomatous vascular pattern.

Diagnosis of BSNS is often confirmed through immunohistochemical staining, revealing positivity for SMA, S-100, calponin, desmin, and, in some areas, myogenin [106, 109–111]. A majority of BSNS cases also exhibit nuclear beta-catenin immunoreactivity [110–114]. Additional immunostains such as factor XIIIa, PAX3, PAX7, PAX8, cytokeratin AE1/AE3, CD34, and TLE1 have also been documented as positive in BSNS [106, 109, 112, 113, 115–120]. Interestingly, Andreasen et al. identified cytoplasmic positivity for STAT6 in three BSNS cases [112]. Conversely, markers such as SOX-10, ER, and progesterone receptor consistently test negative in BSNS, further aiding in its differentiation from other STT entities [106, 111–113, 117, 118].

### PAX3 Fusion Variants in BNSN

In a pivotal study conducted in 2012, Lewis et al. analyzed the karyotypes of two BSNS cases and identified a t(2;4)(q37.1;q31.3) chromosomal translocation, previously unreported in any neoplasms considered in the differential diagnosis [106]. This finding underscores the unique genetic profile of BSNS, particularly its aberrant expression of genes that are key in neuroectodermal and myogenic differentiation, echoing the developmental roles of the PAX3 gene [115]*.*

Wang et al. further advanced this understanding by isolating PAX3-MAML3 cDNA from BSNS tumor mRNA. They inserted this cDNA into a mammalian expression vector and, through transient transcription assays, demonstrated that PAX3-MAML3 fusion significantly upregulates PAX3-driven receptor plasmids, a much more potent effect compared to wild-type PAX3 [115]. This finding suggests that PAX3-MAML3 fusions may play a critical role in BSNS pathogenesis, potentially through disrupting normal cell lineage commitment and activating Notch signaling pathways, which promote tumor growth [103, 109, 112, 117, 120–123]. Interestingly, BSNS tumors with PAX3-NCOA1 and PAX3-FOXO1 fusions have shown morphologic and immunophenotypic characteristics consistent with focal rhabdomyoblastic differentiation [109, 118, 119]. More recent reports from Loarer et al. and Fritchie et al. have identified cases of BSNS with PAX3-WWTR1, PAX3-FOXO1, and PAX3-NCOA2 fusions, while other novel studies have discovered PAX3-FOXO6, PAX3-INO80D, and PAX7-PPARGC1 fusions in BSNS [109, 117, 124••, 125, 126]. 

Despite consistently negative SOX-10 staining in BSNS, gene expression profiling has revealed alterations in a variety of genes involved in neurogenic development, such as NTRK3, ALX1-4, DBX1, GREM1, and NUROG2 [115]. These findings further validate the biphenotypic nature of the tumor. 

Table 1 presents a comprehensive list of gene fusion variants identified in BSNS cases, each verified using one or more of the following methodologies: Sanger Sequencing, NGS, or Real-Time Polymerase Chain Reaction (RT-PCR).

**Table 1:** Identified gene fusion variants in BSNS

Authors (date) / Reference	Gene fusion	Positive case ratio
Le Loarer et al. (2019) / [117]	PAX3-MAML3 PAX3-WWTR1 PAX3-NCOA2	35/44 2/44 1/44
Fritchie et al. (2016) / [109]	PAX3-MAML3 PAX3-FOXO1 PAX3-NCOA1	24/44 3/15 1/15
Wang et al. (2014) / [115]	PAX3-MAML3	19/25
Andreasen et al. (2018) / [112]	PAX3-MAML3	3/3
Muraoka et al. (2023) / [121]	PAX3-MAML3	1/1
Georgantzoglou et al. (2022) / [120]	PAX3-MAML3	1/1
Bell et al. (2022) / [122]	PAX3-MAML3	1/1
Sugita et al. (2019) / [123]	PAX3-MAML3	1/1
Huang et al. (2016) / [118]	PAX3-NCOA1	2/7
Wong et al. (2016) / [119]	PAX3-FOXO1	1/1
Nichols et al. (2023) / [124••]	PAX3-FOXO6	1/1
Vilamontes et al. (2023) / [125]	PAX3-INO80D	1/1
Bhele et al. (2023) / [126]	PAX7-PPARGC1	1/1

Each variant has been verified through one or more advanced genetic analysis techniques: Sanger Sequencing, Next Generation Sequencing, or Real-Time Polymerase Chain Reaction

## Skull Base Chordoma

Chordoma, a rare malignant tumor of the bone, is thought to originate from the remnants of the primitive notochord within the axial skeleton, and typically presents in the skull base and spine [127]. The most common sites for chordomas are the skull base, particularly the clivus and petrous apices, and this review will focus on skull base chordomas (SBC) [128]. SBC are uncommon, constituting less than 0.2% of all intracranial tumors, with an incidence rate of approximately 0.08 per 100,000 individuals annually [129, 130]. They can affect individuals of any age but are rare in children and adolescents, and the incidence is nearly the same across genders for skull base chordomas [131]. Characterized by slow growth and local invasiveness, SBC seldom show lymphatic or hematogenous spread at the time of diagnosis [132]. Treatment poses significant challenges, evidenced by a high local recurrence rate of 53% within five years and 88% over ten years [133]. The overall 5-year survival rate for patients with SBC is about 68% [133]. The preferred treatment approach for these tumors is radical surgical resection, followed by charged-particle radiotherapy, specifically proton beam therapy, targeting the resection cavity and surrounding areas of the initial tumor [134, 135]. However, achieving complete resection of SBC is often challenging due to the proximity of critical neural and vascular structures, including the brain stem, optic nerve/chiasm, and carotid and basilar arteries [136].

### Histopathological Characteristics of SBC

SBC display a distinctive structure composed of large epithelioid cells arranged in cords or clusters. These cells often exhibit cytoplasmic multi-vacuolation, known as physalipherous cells, set within a copious extracellular myxoid matrix [137]. There is a possibility of observing cartilaginous differentiation in these tumors. Despite their malignant nature, the cytological atypia in SBC varies significantly, ranging from low-grade, where tumor cells appear uniform with infrequent mitotic figures, to high-grade, characterized by notable nuclear irregularities and abundant mitoses [138]. SBC demonstrate heterogeneity in terms of atypia, and necrosis is commonly observed [138].

Immunohistochemical analysis shows SBC cells staining positive for cytokeratin, epithelial membrane antigen, S-100 protein, and vimentin, alongside a notable absence of nuclear SMARCB1/INI1 expression [138, 139, 140•]. The most sensitive marker for SBC is brachyury, a nuclear protein indicative of notochordal differentiation [141, 142]. While brachyury is highly specific to SBC, it is noteworthy that in poorly differentiated and dedifferentiated regions of the tumor, brachyury immunoreactivity may be absent [143, 144]. Additionally, a high Ki-67 proliferation index has been noted, and p53 protein accumulation is often observed in the cells of SBC [139, 145–147].

### The Molecular Landscape of SBC

Significant advancements have been made in deciphering the molecular profile of SBC. An extensive review of the literature indicates the benefit of systematically organizing these findings to improve clarity and analytical precision. This systematic approach involves categorizing key areas such as genetic irregularities and chromosomal changes, the impact of microRNAs, aspects related to cell signaling and receptor tyrosine kinases, and the expression of cell adhesion molecules along with the epithelial-mesenchymal transition.

#### Genetic Aberrations and Chromosomal Alterations

Our understanding of the molecular underpinnings in SBC has been significantly enhanced through recent genomic studies, although it remains an area ripe for further exploration. Key insights have been gained into genetic anomalies and chromosomal alterations characterizing these tumors. Notably, duplications in the TBXT gene, deletions in CDKN2A/B, and mutations in genes like LYST, SETD2, and PBRM1 are consistently observed [148••, 149, 150, 151•, 152]. These genetic aberrations are accompanied by chromosomal changes, particularly LOH at 3p and 13q14, which involves the Rb locus [149]. Intriguingly, while LOH at 9p correlates with reduced overall survival, similar changes at 1p, 10q23, or 17p13 do not seem to impact survival rates significantly [145, 152]. A genome-wide single nucleotide polymorphism (SNP) genotyping array analysis reveals that TBXT amplifications, though rare, appear more frequently in sacral than in skull base chordomas [153]. The role of PBRM1 alterations, highlighted by Bai et al., emerges as a significant prognostic factor, suggesting a potential link to the efficacy of anti-programmed cell death protein (PD)-1 checkpoint inhibitors, a connection well-established in other cancer types [148••, 154, 155]. Additionally, partial loss of SMARCB1 through hemizygous 22q deletion or copy number alterations, unlike the complete loss observed in other chordoma subtypes, underscores the importance of the SWI/SNF complex in SBC pathogenesis [148••, 156•, 157, 158].

Genomic profiling has also led to the discovery of recurrent somatic variants, including mutations in MUC4, NBPF1, and NPIPB15, as well as SAMD5-SASH1 gene fusion [159]. Of particular interest is the identification of a germline functional SNP, rs2305089, in the T gene, strongly linked to SBC occurrence. The duplication of the T gene, encoding brachyury, is observed in familial SBC and sporadic cases, offering insights into tumor development [160, 161]. Bell et al.'s RNA sequencing analysis further enriches our understanding by highlighting five upregulated genes (T, LMX1A, ZIC4, LHX4, and HOXA1) as potential biomarkers [162]. Moreover, the presence of TP53 mutations, primarily in dedifferentiated components of SBC, hints at the role of the p53 pathway in the tumor's pathology [163, 164]. A unique molecular characteristic of SBC is the loss of H3K27me3 in dedifferentiated chordomas, a marker retained in sacral chordomas [137]. Additionally, MGMT promoter methylation observed in recurrent clival chordomas and the absence of IDH1 and IDH2 mutations, commonly seen in conventional chondrosarcomas, further differentiate SBC at the molecular level [165–167].

#### Role of microRNAs

The molecular landscape of SBC is further illuminated by studies on microRNAs (miRNAs), which play critical roles in cancer initiation and progression [168]. Kuang et al. discovered a significant decrease in miRNA 10a and 125a in SBC [169]. These antitumor miRNAs are inhibited by the ADAR gene, which is found to be overexpressed in this condition [169]. Bayrak et al.'s microarray analysis of fresh SBC samples identified key miRNAs such as miR-31, miR-140-3p, miR-148a, and the miR-221/222 cluster [170]. Notably, hsa-miR-31 has been found to induce apoptosis in chordoma cells and to modulate the expression of c-MET and radixin, offering potential therapeutic targets [170].

#### Cell Signaling and Receptor Tyrosine Kinases

The recurrence and progression of SBC are closely linked to cell signaling pathways. High expression levels of TGFalpha, bFGF and fibronectin correlate with increased recurrence rates [171]. Elevated levels of c-MET and epidermal growth factor receptor (EGFR) are often noted in SBC samples, while the expression of c-Erb-b2 (HER2/neu) shows variability [172]. Shalaby et al. reported that a significant proportion of SBC cases exhibit high-level EGFR copy number gains, with a majority expressing total EGFR, suggesting a potential avenue for EGFR-targeted therapies [173]. Immunohistochemical analysis in a study of 21 SBC cases revealed the presence of receptor tyrosine kinases like HER2, KIT, EGFR, and PDGFR-β [174]. The detection of phosphorylated isoforms indicative of tyrosine kinase activity, such as p44/42-mitogen-activated protein kinase, Akt, and STAT3, further underscores the potential of targeting these pathways in treatment strategies. The discovery of high levels of phosphorylated PDGFR in SBC has already influenced the adoption of novel chemotherapeutic agents [175, 176].

#### Expression of Cell Adhesion Molecules and Epithelial-Mesenchymal Transition

The role of cell adhesion molecules and the process of epithelial-mesenchymal transition (EMT) in the pathology of SBC has garnered considerable attention. Research has documented the expression of molecules like E-cadherin, beta-catenin, gamma-catenin, and neural cell adhesion molecule within these tumors [177, 178]. A particularly interesting observation is the inverse correlation between E-cadherin and N-cadherin expression in clival chordomas, suggesting a significant role for EMT [179]. EMT is a critical process where epithelial cells lose their cell-cell adhesion properties and gain migratory and invasive capabilities, transitioning into mesenchymal cells. This transition is pivotal in SBC invasiveness and metastatic potential. Zhang et al. have identified a partial EMT program in SBC cells and demonstrated the potential effectiveness of the TGF-betaR1 inhibitor Y-L13027 in attenuating tumor growth [180••]. This inhibitor targets the p-EMT pathway, emphasizing the role of ZEB2 and its association with the p-EMT marker TGFbeta1. These findings highlight the importance of the EMT process in the aggressiveness of SBC and suggest new avenues for therapeutic intervention.

Further studies have delved into the role of local invasiveness in SBC. High levels of matrix metalloproteinases (MMP-1, MMP-2) and related proteins like tissue inhibitor of MMP-1/2, cathepsin-B, and urokinase plasminogen activator (uPA) have been observed [181]. These molecular characteristics correlate with tumor infiltration into the host bone and have been linked to a worse prognosis in primary and recurrent SBC. The elevated expressions of MMP-1 and uPA, in particular, may serve as biomarkers for aggressive disease and provide valuable prognostic information.

### Advances in the Treatment of SBC

Compared to the three mesenchymal STTs previously discussed, recent clinical trials targeting SBC have yielded promising results, particularly through the use of receptor tyrosine kinase inhibitors, checkpoint inhibitors, and CDK4/6 inhibitors. It is noteworthy that these trials typically include patients diagnosed with chordoma, regardless of its specific anatomical location, and those with locally advanced, unresectable, or metastatic disease. Table 2 provides a carefully selected overview of pharmacological clinical trials targeting SBC.

**Table 2:** Curated selection of pharmacological clinical trials targeting SBC

NCT ID	Status	Phase	Intervention	Target
NCT01175109	Unknown Status	I	Imatinib + LBH589 (Panobinostat)	PDGFR/Histone deacetylase inhibitor
NCT03190174	Completed	I/II	Nivolumab + ABI-009 (Nab-rapamycin)	PD-1/mTOR
NCT05407441	Recruiting	I/II	Tazemetostat + Nivolumab + Ipilimumab	EZH2/PD-1/CTLA-4
NCT05286801	Recruiting	I/II	Tiragolumab + Atezolizumab	TIGIT/PD-L1
NCT05041127	Recruiting	II	Cetuximab	EGFR
NCT06140732	Recruiting	II	Apatinib + Camrelizumab	VEGFR/PD-1
NCT03242382	Recruiting	II	Palbociclib	CDK-4/6
NCT04416568	Recruiting	II	Nivolumb + Ipilimumab	PD-1/CTLA-4
NCT05519917	Not yet Recruiting	II	Afatinib	ErbB-family
NCT03623854	Active, not Recruiting	II	Nivolumab + Relatlimab	PD-1/LAG-3
NCT03083678	Active, not Recruiting	II	Afatinib	ErbB-family
NCT02601950	Active, not recruiting	II	Tazemetostat	EZH2
NCT03110744	Completed	II	Palbociclib	CDK-4/6
NCT00150072	Completed	II	Imatinib	PDGFR
NCT00464620	Completed	II	Dasatinib	PDGFR
NCT04042597	Unknown status	II	Anlotinib Hydrochloride	VEGFR, FGFR, PDGFR, c-kit

*PDGFR* platelet-derived growth factor receptor; *PD-1* programmed cell death protein 1; *mTOR* mammalian target of rapamycin; *EZH2* enhancer of zeste homolog 2; *CTLA-4* cytotoxic T-lymphocyte associated protein 4; *TIGIT* T-cell immunoreceptor with Ig and ITIM domains; *PD-L1* programmed death-ligand 1; *EGFR* epidermal growth factor receptor; *VEGFR* vascular endothelial growth factor receptor; *CDK-4/6* cyclin-dependent kinase 4/6; *LAG-3* lymphocyte activation gene 3; *FGFR* fibroblast growth factor receptor

#### Receptor Tyrosine Kinase Inhibitors

Extensive research has revealed that SBC often exhibits active tyrosine kinase receptors, particularly MET, PDGFR, EGFR, HER2 (ERBB2), KIT (SCFR), and VEGFR (KDR) [175, 182]. This discovery has prompted investigations into receptor-targeted therapies for chordoma patients, specifically those whose tumors express such targets. Clinical trials have been conducted with various drugs such as lapatinib and erlotinib (targeting EGFR and HER2), imatinib and dasatinib (targeting PDGFR), and sorafenib and sunitinib (targeting VEGFR) [176, 183–187]. A retrospective study of 46 metastatic chordoma cases, all PDGFR-β positive, found a median progression-free survival (PFS) of 9.9 months [184]. During a median observation period of 24.5 months, stable disease (SD) was noted in 34 out of 46 patients as per RECIST 1.0 criteria, without any partial or complete responses. This aligns with earlier phase II trial results involving 50 advanced chordoma patients treated with imatinib, showing a median PFS of 9 months and SD in 70% of the cases [183]. The low response rates in these trials, despite high PDGFR-β expression, hint at the possibility of exploring alternative pathways for treatment beyond PDGFR-β. Consequently, a combination therapy of imatinib and a histone deacetylase (HDAC) inhibitor is currently being assessed in recurrent chordoma patients (NCT01175109). In a phase II clinical trial involving lapatinib, 18 patients with advanced, progressing chordoma were examined [186]. The study assessed the expression and activation of EGFR through immunohistochemistry and/or phosphoarrays. Results showed partial responses in six patients (33.3%) and SD in seven patients (38.9%), based on the Choi criteria. The median PFS observed in this study was 6 months. Conversely, other EGFR inhibitors have shown promising responses, indicating their potential efficacy against these tumors [188, 189]. For example, afatinib, another EGFR inhibitor that targets multiple ErbB family members, has shown promise in preclinical studies and is currently being evaluated in two separate phase II trials for EGFR-expressing chordoma patients (NCT03083678, NCT05519917) [190, 191••]. Scheipl et al. conducted a drug screening involving 133 approved anticancer drugs, both as standalone treatments and in combination with EGFR inhibitors (EGFRis; such as afatinib and erlotinib) [192••]. They discovered that combining crizotinib, panobinostat, and doxorubicin with EGFRis presents a promising therapeutic approach. Specifically, the HDAC inhibitor panobinostat displayed a moderate synergistic effect when combined with afatinib. Although the study did not reveal significant success for these drugs as single-agent therapies in solid tumors, including chordoma, it suggested their potential effectiveness in combination therapies and multi-target inhibition strategies [193]. Studies also highlight the effectiveness of combined HDAC and PDGFR inhibition in addressing PTEN disruptions in chordoma [194]. Moreover, a phase II clinical trial with sorafenib, which has shown in vitro activity against VEGFR and PDGFR, indicated a longer PFS compared to imatinib [195]. Apatinib, another VEGFR inhibitor, was tested in a phase 2 study in China, with promising results including an objective response in one patient and a median PFS of 18 months [196••].

#### Immunotherapeutic Strategies

Immunotherapy has made significant progress in cancer treatment, especially in targeting immune checkpoint molecules like PD-1 and cytotoxic t-lymphocyte associated protein 4 (CTLA-4) [197]. PD-1 inhibitors such as nivolumab and camrelizumab enhance the immune response against tumors by blocking PD-1’s interaction with the respective ligands on tumor cells [198]. Studies on PD-1 and programmed death-ligand (PD-L)1/PD-L2 expression in chordoma have yielded mixed results, limited by small sample sizes [199, 200]. A study by Mathios et al. specifically found that while chordoma cells did not express PD-L1, this protein was present in macrophages and T cells [200]. Later research, which examined 78 tissue samples, delved deeper into the expression of PD-L1 and its association with the clinical profiles of chordoma patients [199]. Contrary to Mathios' findings, these subsequent studies indicated positive PD-L1 expression in the tumor cells themselves, and this expression correlated with a worse prognosis for chordoma patients [201]. CTLA-4 is targeted by ipilimumab, the first FDA-approved therapy for immune checkpoint blockade, which plays a vital role in deactivating T-cell-based immune attacks [202]. TIGIT, another immune checkpoint molecule, is targeted by tiragolumab to enhance antitumor responses [203]. Various clinical trials are currently assessing the efficacy of these immune checkpoint inhibitors in chordomas, including studies on nivolumab (NCT03623854, NCT03190174, NCT04416568), camrelizumab (NCT06140732), atezolizumab (NCT05286801), ipilimumab (NCT04416568), and tiragolumab (NCT05286801), either as single agents or in combination with other therapies. One notable combination therapy includes nivolumab, ipilimumab, and the enhancer of zeste homolog 2 (EZH2) inhibitor tazemetostat (NCT05407441). EZH2, a component of the PCR2 polycomb repressive complex, is implicated in oncogenesis, and agents targeting it have shown potential in inducing tumor regression and enhancing radiation sensitivity in SMARCB1/INI1-deficient tumors, including chordomas [204, 205].

#### CDK4/6 Inhibitor

The frequent deletion of the p16 (CDKN2A) tumor suppressor gene in SBC cell lines and patient biopsies points to a universal activation of the CDK4/6 pathway in these tumors [148••, 149, 150, 151•, 152]. Studies using patient-derived chordoma cell lines have demonstrated that abnormal CDK4/6 activity, resulting from p16 loss, can be effectively targeted by the CDK4/6 inhibitor palbociclib, leading to a reduction in tumor cell proliferation and growth [191••, 206•, 207, 208]. Currently, two phase II clinical trials are underway to evaluate the effectiveness of palbociclib in patients with advanced/metastatic chordoma who are not eligible for standard treatments (NCT03110744, NCT03242382).

## Future Directions and Conclusions

The *fifth edition of the World Health Organization Classification of Head and Neck Tumors* marks a significant advancement in the categorization and understanding of STTs. This classification not only introduces a plethora of new, well-defined, and emerging STTs but also highlights the diverse range of mesenchymal entities within this category. Mesenchymal STTs are particularly challenging to manage due to their rarity, often indolent growth patterns, and the subtlety or non-specificity of their presenting symptoms. Therefore, a comprehensive understanding and approach to these complexities are paramount for the effective management and treatment of STTs. A cornerstone in the primary characterization and diagnosis of most STTs lies in their histological features. This traditional diagnostic method is significantly enhanced by the integration of targeted immunohistochemical and molecular testing. Adding to these developments, a novel classification system based on methylation patterns has been recently introduced for STTs [209••]. This innovative approach delineates four distinct molecular subtypes and includes sinonasal undifferentiated tumors. The utility of these methylation-based assays offers an intriguing possibility for application in the diagnostic classification of mesenchymal STTs.

The utilization of advanced molecular genetic techniques, such as LOH, FISH, CGH, and RT-qPCR has led to the identification of various chromosomal imbalances, alterations in key tumor suppressor genes like APC and TP53, and the variable expression of oncogenes such as c-myc and c-kit in STA. Moreover, the intricate network of growth factors, including VEGF, FGF, and TGFb1, has been found to play a substantial role in STA's angiogenesis and development, while the influence of hormonal dynamics on STA pathogenesis remains an area of active investigation. For SGP, the identification of CTNNB1 gene mutations leading to beta-catenin accumulation in the nucleus and its effect on the Wnt signaling pathway represents a pivotal discovery. Similarly, the detection of PAX3 fusion variants in BSNS has significantly altered the perception of its genetic framework. In the case of SBC, recent investigations have revealed a complex genetic landscape with notable findings including TBXT gene duplications, CDKN2A/B deletions, mutations in genes like LYST, SETD2, PBRM1, and the loss of heterozygosity at critical chromosomal locations. The high expression of growth factors and receptor tyrosine kinases in these pathways suggests new therapeutic targets, currently being tested in clinical trials. Furthermore, research into EMT and the role of cell adhesion molecules such as E-cadherin and N-cadherin has provided insights into the invasiveness and metastatic potential of SBC.

In contrast to these recent advancements, the rare nature, scarcity of adequate in vitro and in vivo models, and the inherent heterogeneity of STTs underscore the ongoing challenges in understanding and targeting these conditions. To address these issues, patient-derived organoids (PDOs) have garnered significant interest as effective models [210]. They offer accurate representations of patient tumors and are more efficient in terms of initiation time, cost, and overall efficiency than patient-derived xenografts. This approach is now being actively investigated in chordoma research. For instance, a recent study successfully created chordoma PDOs from five different patients, using them to screen various drugs for potential personalized repurposing [211•]. In other types of cancer, PDOs have been shown to closely replicate patient drug responses and have been employed in personalized treatment strategies [212–214]. The slow growth rate of chordoma tumors provides a significant time window to develop and refine protocols for establishing and validating chordoma PDOs, particularly for patients at high risk or those experiencing relapse. This could facilitate the identification of effective drug repurposing strategies within a clinically relevant timeframe for treatment decisions.

Viral infections play a crucial role as an etiological factor in the development of various tumors, making them a compelling area of future research in the pathogenesis of mesenchymal STTs. For example, recent findings highlight the prevalence of oncogenic viruses in chordomas, demonstrating the variable presence of genomic DNA from viruses such as BPV19, EBV, and HHV7 [215]. Additionally, the advent of innovative therapies, particularly CAR T-cell therapy, presents a promising avenue, especially in the treatment of SBC [216•].

In summary, the challenge to fully unravel the complexities of mesenchymal STTs and to develop effective treatment strategies remains a work in progress. Embracing a multidisciplinary approach, which integrates innovative technologies and emerging therapies, is crucial in this endeavor. This comprehensive strategy is key to unlocking new possibilities in the management and treatment of these challenging tumors, paving the way for more effective and personalized therapeutic options in the future.

## Data Availability

No datasets were generated or analysed during the current study.
